# Genetic factors predisposing to bronchopulmonary dysplasia. A pilot study by exome sequencing and pathways analysis

**DOI:** 10.1186/1824-7288-41-S1-A42

**Published:** 2015-09-24

**Authors:** Marco Somaschini, Chiara Di Resta, Chiara Volonteri, Emanuela Castiglioni, Silvia Bonfiglio, Dejan Lazarevic, Davide Cittaro, Elia Stupka, Maurizio Ferrari, Paola Carrera

**Affiliations:** 1 Unit of Genomics for Diagnosis of Human Pathologies, Division of Genetics and Cell Biology, IRCCS Ospedale San Raffaele, Milano, Italy; 2 Laboratory of Clinical Molecular Biology, IRCCS Ospedale San Raffaele, Milano, Italy; 3 Vita-Salute San Raffaele University, Milano, Italy; 4 Centre for Translational Genomics and Bioinformatics, IRCCS Ospedale San Raffaele, Milano, Italy; 5 Unit of Neonatology, Clinica Sant'Anna, Lugano-Sorengo 6924, Switzerland

## Background

Bronchopulmonary Dysplasia (BPD) is a multifactorial disease with a significant genetic component. Twin studies indicate that heritability of BPD is estimated at 53 to 79% [1]. Association studies have identifiedseveral potential candidate genes encoding components of innate immune and antioxidant defenses, mechanisms of vascular and lung remodeling, matrix remodeling proteins, surfactantproteins [2,3]. We planned a prospective multicentre study aimedto identify rare genetic variants contributing to the BPD phenotypeby exome sequencing using next-generation sequencing (NGS) technology. 

## Materials and methods

26 unrelated newborns with a clinicaldiagnosis of severe BPDaccording with NIH Consensus Criteria[4]were selected among a collected cohort of 366premature neonates of European origin with gestational age ≤ 32 wfrom 12Italiancenters. Genomic DNAwas extracted from peripheral blood and exome sequencing was carried out on an IlluminaHiSeq2000 platform. In order to identify potentially interesting variants related to BPD pathogenesis, we adopted two different strategies: 1) Candidate genes previously associated with BPD in association studies 2) Prioritization analysis based on pathways potentially involved in the pathogenesis of BPD (ToppGene Prioritization tool).

## Results

**Figure 1:**
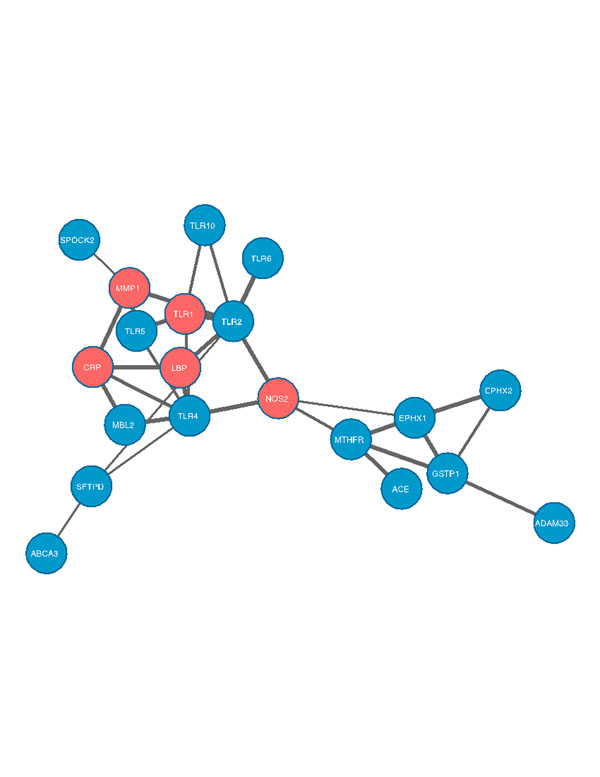
network including genes previously associated with BPD susceptibility and the 5 genes highlighted by toppGene analysis

1) Candidate genes: we identified a total of 61variants in 19 candidate genes previously associated with BPD and confirmed them with Sanger; 31 are commonpolymorphism, 25 are rare and classified as dbSNPrs with a MAF <0.05and 6 are novel. Considering all the variants, the most mutated genes are those belonging to the TLR-family (TLR10, TLR1, TLR4), to oxidative stress-related genes (EPHX2, MTHFR, EPHX1)and to surfactant metabolism genes (SFTPD, ABCA3).2) Prioritizationanalysis: we decided to focus first on the list of the top 5 genes: TLR1, MMP1, NOS2, CRP and LBP. To evaluate the possible interaction between candidate genes previously associated with BPD and showing variants in our sample(ABCA3, SFTPD, SPOCK2, ACE, MTHFR, EPHX1, EPHX2, TLR5, TLR10, TLR1, TLR6, TLR4, GSTP1, MBL2, TLR10, TLR2) and the top 5 genes (NOS2, TLR1, MMP1, CRP, LBP) highlighted with prioritization analysis we usedString 9.122. The results allow the possibility of a networking with a main focus ongenes involved in inflammation (figure 1)[5].

## Conclusions

In consideration of the results obtained in this pilot study, we canconclude that our approachmay be interesting to initiate the dissectionof genetic pathogenesis of BPD.Our study indicates that genes regarding inflammatory response and tissue remodeling may be relevant in BPD pathogenesis. These preliminary resultsneed to be confirmed and may contribute in improving knowledge of pathogenesis of BPD and targeting therapeutic interventions.
